# Constraints in temperature adaptation reinforce differences in thermal niche between mesophilic and psychrotolerant *Bacillus cereus* group species

**DOI:** 10.1098/rspb.2025.1184

**Published:** 2025-07-30

**Authors:** Hugh White, Michiel Vos, Daniel Padfield, M. D. Sharma, Samuel K. Sheppard, Ben Raymond

**Affiliations:** ^1^ Nuffield Department of Population Health, University of Oxford, Oxford OX3 7BN, UK; ^2^ Medical School, University of Exeter, Penryn TR1 3HD, UK; ^3^ Faculty of Environment, Science and Economy; Ecology and Conservation, University of Exeter, Penryn TR10 9FE, UK; ^4^ Faculty of Environment, Science and Economy; Ecology and Conservation, University of Exeter, Penryn TR10 9FE, UK; ^5^ INEOS Oxford Institute, University of Oxford, Oxford OX1 3RE, UK; ^6^ Faculty of Environment, Science and Economy; Ecology and Conservation, University of Exeter, Penryn TR10 9FE, UK

**Keywords:** *Bacillus cereus sensu lato*, niche differentiation, psychrotolerance, experimental evolution, thermal adaptation, adaptive constraint

## Abstract

Experimental evolution has demonstrated that mesophilic microbes readily adapt to increases in temperature. However, many microbes are psychrotolerant and resistant to cold, which is associated with physiological specializations, suggesting constraints in thermal adaptation. We hypothesized that constraints would limit adaption differently in a mesophilic species (*Bacillus thuringiensis*) compared with its psychrotolerant relative *B. mycoides*—with adaptation at cooler temperatures and adaptation at higher temperatures being constrained in each species, respectively. To test this hypothesis, we imposed 140 generations of selection at temperatures at and below the optimum for productivity for both species. The fitness and thermal performance of evolved bacteria showed ancestral thermal niche plays a role in thermal adaptation over this time scale, in support of our hypothesis of adaptive constraints. Temperature-dependent trade-offs appeared common in *B. mycoides*, with fitness gains associated with decreases in operational niche width; fitness gains at one temperature caused a decrease in the range of temperatures that the bacterium showed appreciable growth. Genome resequencing showed that variation in mutation supply and selection strength could not explain temperature-dependent responses to selection. Importantly, metabolic theory only held true for mesophilic *B. thuringiensis,* showing abundant but less studied psychrotolerant species could follow different adaptive trajectories.

## 1. Background

Microbial responses to temperature shifts will have great importance as climate change worsens, changing the function of ecosystems and aggravating the risk of emerging pathogenicity [1,2]. Understanding phenotypic changes and genetic mechanisms of thermal adaptation is therefore essential to predict future ecological responses. The metabolic theory of ecology provides a framework for predicting and understanding patterns of thermal adaptation: because enzymes are more efficient at higher temperatures, thermodynamic constraints may be lower at higher temperatures (‘hotter-is-better’ or ‘HIB’) [3–6]. Additionally, high performance across wide temperature ranges may be challenging and may trade off against peak performance in a ‘specialist-generalist trade-off’ [4,7,8]. Finally, if thermodynamic constraints are weak or non-existent, because thermal performance is highly polygenic, organisms might be able to maximize their performance under any thermal conditions, a ‘perfect adaptation’ hypothesis [9,10].

Much of our understanding and testing of these ideas in a microbial context has been based on studies using mesophiles (i.e. bacteria with optimum temperatures of 25–35°C that cannot grow at 7°C and below) [11]. Mesophiles can rapidly adapt to higher temperatures, suggesting small thermodynamic constraints at their upper thermal range [3,12–14]. Green algae, for instance, can adapt to high temperature after only 100 generations of selection [15]. However, the same may not be true of extremophile bacteria, which may experience different constraints to thermal adaptation. For example, thermophilic prokaryotes are less able to adapt to higher temperatures than mesophiles [16]. Moreover, adaptation to cold environments appears heavily constrained by physiology and requires specialized adaptations [17]. The enhanced performance of mesophiles at high temperatures may therefore not be representative of all bacteria.

The thermal constraints of psychrotolerant microbes—those adapted to lower temperatures than mesophiles—are little studied. Unlike true cold specialist psychrophiles, psychrotolerant species are ‘cool’ adapted, i.e. able to grow at lower temperatures than mesophiles (<10°C) but with thermal optima above 20°C [11,18]. These strains are common in temperate soils, playing key ecological roles in nutrient cycling and promoting plant growth [19,20]. Psychrotolerant strains have many adaptations for growth at cool temperatures, including changes in membrane composition, altered translation and transcription machineries [21]. Enzyme comparisons show that high conformational flexibility allows a greater efficacy at low temperatures, sometimes with a cost to enzyme thermal stability [22,23]. This suggests high levels of thermal specialization that might impose limits on adaptation of mesophiles to psychrotolerant conditions. This physiological evidence is supported by phylogenetic analysis. For example, bacterial clades within the *Bacillus cereus sensu lato* complex have distinct thermal niches [11,24,25] with thermal preferences reliably associating with phylogeny and other physiological traits [26,27] indicating that individually evolving lineages cannot readily switch thermal preferences. Additionally, while studies investigating adaptation to temperature under the optimum exist, this is relatively understudied compared with high temperature adaptation studies and often has ignored especially low temperatures [28]; furthermore, fluctuating temperature studies have suggested that some species—but not others—may adapt asymmetrically to high temperatures more than to low temperatures [29–31].

In this study, we test the hypothesis that bacterial species are subject to strong constraints which limit adaptation to novel thermal niches. While previous work has shown that mesophilic microbes can readily adapt to higher temperatures [8,16], we predicted that this would not hold true for psychrotolerant bacteria. In addition, we predicted that mesophiles are constrained in their ability to adapt to temperatures below their thermal optima, due to a lack of specific physiological features needed to control cell membrane composition, enzyme structure and translation machineries [21]. We conducted experimental evolution using a mesophilic *B. thuringiensis* strain [32] and a psychrotolerant *B. mycoides* [11] each belonging to the *B. cereus* (*Bc*) group [33]. After approximately 140 generations of adaptation to temperatures close to and far below thermal optimum, we used a three-step analytical approach. Competition assays were used to test for fitness changes via direct competition with the strain’s ancestor; thermal performance curves of growth rate and productivity were used to characterize the phenotypic nature of fitness changes and test hypotheses associated with metabolic theory; and single nucleotide polymorphism (SNP) variant calling was used to identify genetic changes that emerged during experimental evolution.

## 2. Methods

### (a) Generation time estimation and mutant construction

Two strains with different thermal phenotypes were used in experiments; the mesophilic *B. thuringiensis* BGSC 4D7 (a *Cry* null variant cured of toxin producing plasmids) sourced from the Bacillus Genetic Stock Centre and the psychrotolerant *B. mycoides* BR D096i sourced from the field in the UK [34,35]. Generation time and time to stationary phase for each strain at two experimental temperatures (15°C and 30°C) were verified through experimental growth in Brain-Heart Infusion (BHI), an amino-acid-rich non-sporulating medium. BHI represents growth within a host or cadaver and *Bc* strains are well-adapted to use it as a nutrient source [35,36]; this minimizes the selective pressure exerted on lineages by the media, allowing us to isolate temperature-specific adaptations. Plating of samples throughout the experiment and logistic growth modelling using optical density (OD_600_) data in GrowthCurver [37] indicated that generation times varied by temperature but not by strain, with around 9 and 3 generations per day at 30°C and 15°C, respectively.

Low-cost rifampicin-resistant mutants were selected to provide marked competitors in selection and fitness experiments [38]. Multiple independent mutants were isolated by plating dense suspensions of overnight cultures of each strain on Luria Broth (LB) agar containing 50 µg ml^−1^ rifampicin. Competition experiments with ancestors at 15°C and 30°C were used to identify mutants with minimal fitness costs and clones with the smallest fitness differences relative to ancestors were chosen for experiments.

### (b) Experimental evolution

Experimental lineages were founded by inoculating samples of ancestral strains into 5 ml of BHI in individual universal glass vials (BHI; Thermo Scientific). We used four genetic backgrounds, consisting of wildtype (WT) ancestors and rifampicin-resistant mutants of *B. thuringiensis* and *B. mycoides*. These were grown at 30°C and 200 r.p.m. for 24 h, then 50 µl of these cultures were used to inoculate fresh vials of BHI. This was repeated 10 times to produce five replicate lineages for each experimental temperature of 15°C and 30°C. Transfers using 50 μl of media occurred every 24 h in the 30°C treatment and every 72 h in the 15°C treatment, as preliminary experiments indicated that stationary phase was reached at these points. Samples of each lineage were stored at −80°C every three transfers, and lineages were moved through 21 transfers; as mathematical estimates suggest a maximum number of doublings per transfer as 6.64, we calculate that approximately 140 generations passed in this experiment. Lineages are labelled by species, selection regime and lineage (e.g. Bt301, Bm151, etc.).

### (c) Competition assays

Changes in fitness following experimental evolution were determined using competition experiments in which ancestor strains were competed against the evolved lineages. Frozen glycerol stocks from each selected line and ancestral line were inoculated into fresh BHI and grown overnight at 30°C. Thereafter, 50 µl was transferred into 5 ml of fresh media without rifampicin as above and grown at either 30°C for 24 h or 15°C for 72 h at 200 r.p.m. to acclimate strains to the competition conditions and to ensure similar physiological states in competitors [39]. Competition assays used six replicates for each independently evolved lineage. The ratio of resistant strain to acclimated strain in the starting inocula varied depending on which was the acclimated strain; when the acclimated strain was rifampicin resistant, the ratio used was 1 : 9 in favour of the WT on the assumption that acclimation would boost fitness of the resistant strain. In contrast, acclimated WT strains were mixed 50 : 50, and the exact initial ratio was confirmed by replica plating 100 colonies from each mixture on antibiotic-free and selective LB agar. Initial ratios and population densities were also checked by replica plating on selective and antibiotic-free LB agar.

Measures of fitness rely on the Malthusian parameter, defined as the log ratio of final density divided by initial density [40]. *Relative* performance relies on comparing Malthusian parameters of different strains, either as the difference between them (the selection rate constant) or their ratios (the relative fitness) [38]. As relative fitness is very sensitive to sampling error when strains differ greatly in their Malthusian parameters, we compared strain performance using the selection rate constant. A positive selection rate constant indicates a higher performance in the evolved lineage compared with the ancestral strain [35,38]. For simplicity, we refer to the selection rate constant as ‘fitness’ in §3.

We tested for effects of selection regime and competition temperature on fitness using mixed effect models in R v. 4.1.2 packages (*lme4* and *lmerTest*) for each species separately [41]. Fixed effects were selection regime (ancestral strain, 15°C or 30°C), competition temperature (15°C or 30°C) and their interactions, with lineage as a random intercept accounting for non-independence of fixed effects. Initially, analyses were conducted separately by species and resistance phenotype; when resistance phenotype was seen not to exert a significant effect on fitness, lineages were separated by species only. Significance testing used model simplification, likelihood ratio tests and planned *post hoc* comparisons between each evolved lineage and the ancestral strain at each competition temperature. Additionally, differences between individual lineages and respective ancestors were determined by refitting models as one-way ANOVAs using lineage as a factor. A general linear model was produced with ancestral fitness at 15°C as the intercept and planned *post hoc* contrasts were used to compare selected lines to the ancestor at each competition temperature. In all models, graphical analyses were used to verify model assumptions.

Since adaptation to warmer temperatures has been associated with changes in cell size, we measured *B. thuringiensis* and *B. mycoides* cells from lineages evolved at 30°C alongside their ancestors. These experiments used standard outgrowth conditions in 5 ml 2% BHI, as above. Following incubation, 1 ml overnight culture was spun down (>1 min at 17 000 r.p.m.) and cells resuspended in saline prior to staining (incubation at 4°C in dark) with BacLight bacterial viability and counting staining kit. Images were acquired using a confocal fluorescence microscope (Leica AF6000) set up as follows: objective × 63 plan apochromatic; SYTO9 excitation wavelength 488  nm, emission wavelength 493−547 nm; propidium iodide excitation wavelength 552  nm and emission wavelength 567−725 nm. Analysis of images used Leica software (LAS X version 3.5.1.18803) to measure cells over five independent fields of view per strain. Dimensions were recorded as pole-equator lengths in μm for a total of 30 cells/strain in focus and planarity. Cell volume was calculated assuming an ellipsoid form (cell vol = 4⁄3 *π* × *ρ*/2× *ϵ*) where *ρ* is the polar long axis and *ϵ* is the equatorial short axis.

### (d) Thermal profiling

We calculated thermal performance curves for both growth rate and productivity as follows. Each WT strain and evolved lineage from each selection regime (15°C or 30°C) was inoculated into 1 ml BHI from frozen glycerol stocks and grown at 30°C for 24 h in 24 well plates. Two hundred microlitres of each evolved lineage and ancestor strain was inoculated into 800 µl of fresh media in a well in a 24 well plate. Three replicates for each evolved lineage and six replicates for each ancestral strain were created in a given plate. This was repeated to create 10 plates, each for a different experimental temperature.

Each lineage or ancestral strain was grown at an experimental temperature for 24 h to allow acclimation; as we were interested in differences in response between strains and we gave each strain approximately the same number of generations to acclimate, the need for temperature-specific acclimation times was negligible. Optical density for each replicate at each temperature was assessed using a Varioskan Flash plate reader (Thermo Fisher Scientific Inc.), and replicates were diluted appropriately to an OD_600_ of 0.045 in a new plate. Plates were incubated for 24 h at a range of temperatures between 12°C and 39°C in intervals of 3°C. Optical density readings (OD_600nm_) were taken every 10 min for 24 h. We used the R package *Growthcurver* [37] on each replicate to fit logistic growth models and obtain growth parameters, specifically exponential growth rate (*r*) and productivity (auc_E, or empirical area under the growth curve, encompassing growth rate and carrying capacity) [35,42]. The full dataset up to 39°C was used to calculate growth parameters.

Thermal performance curves for each species at each selection temperature were modelled using *rTPC* and *nls.multstart* packages in R [43] using the Sharpe–Schoolfield model for high temperature inactivation [8]. This model was used to increase parsimony; the Sharpe–Schoolfield model is commonly used, but assumes complete inactivation of the rate-determining enzyme at low temperatures; as we did not observe this in our study, a model ignoring low temperature enzyme inactivation was more appropriate [44,45].

There was high variability in growth rate and productivity amongst the replicates at 39°C in both species and at 36°C in *B. mycoides*; as a result, these data were not used to fitTPCs. The parameters extracted from TPCs were *Topt* (optimum growth temperature), maximum growth rate/productivity (*Bpk*/*Ppk*), *Wop* (operational thermal niche width) and selection temperature (15°C, 30°C and the ancestral control). *Wop* is defined as the difference between *Topt* and the temperature at rise of curve where growth rate is half of *Bpk*/*Ppk* [8] as species typically experience temperatures below their optimum and our TPCs typically do not cover a sufficient temperature range to estimate the full niche width.

Significance tests were conducted using linear models; in these, *Bpk* or *Ppk* was the response variable, with *Topt* or *Wop* and selection temperature as explanatory variables. Planned *post hoc* contrasts were also used to compare the growth rate and productivity of evolved lineages to their ancestor. Model testing for a lack of constraint to bacterial thermal adaptation (the ‘perfect adaptation’ hypothesis; [8]) used selection regime and species as explanatory variables; to maximize confidence and simplify analysis, the models were also tested for each species separately using only selection temperature as an explanatory variable. *p*-Values were determined using likelihood ratio tests.

### (e) Whole-genome sequencing and comparison of selected and control lines

To obtain DNA for sequencing, clonal stocks of the ancestral strains and frozen samples from the end of experimental evolution (representing mixed populations) were inoculated directly into BHI media and grown overnight at 30°C. Short-read sequencing was conducted on ancestral and evolved strains using the company MicrobesNG, who conducted the DNA extraction and sequencing (Birmingham, UK; http://www.microbesng.uk). The ancestral *B. mycoides* strain was also subjected to long-read sequencing; DNA was extracted using the DNEasy Blood and Tissue Kit and sequenced using the Oxford Nanopore sequencing platform MinION [46]. An annotated assembly of *B. thuringiensis* (*kurstaki* HD73) was available through the National Centre for Biotechnology Information (NCBI) (ASM33875v1; Genbank accession = GCA_000338755.1).

To ensure the quality of the *B. mycoides* ancestral genome, trimming was conducted with Porechop [47]. Different types of hybrid assemblies of the ancestral *B. mycoides* genome [48] were produced using Flye v.2.9 [49]. Sequences were polished using Medaka v.1.5.0 [50]. Two Flye methods were used: -meta and -asm-coverage-50x. The -meta approach produced an assembly of nine contigs, five of which were complete, while the -asm-coverage-50x approach produced five complete contigs. We used the -asm-coverage-50x *B. mycoides* assembly for SNP variant analysis. This was annotated using KBase [51] and found to contain 25 946 annotations. Quality was assessed using the Quality Assessment Tool (QUAST) [52] and the Benchmarking Universal Single-Copy Orthologs (BUSCO) tool [53] to assess genome structure and genome content, respectively.

In order to produce an accurate reference genome of the *B. thuringiensis* ancestral strain, we mapped the short read Illumina data from MicrobesNG to the reference genome *B. thuringiensis kurstaki* HD73 (ASM33875v1) using the Geneious Prime mapper (v. 2022.1.1) to add annotations to the reference and identify variants already present in the ancestor [54].

### (f) Variant calling

Through MicrobesNG, we carried out short read Illumina sequencing of evolved lineages from both species. Illumina reads were mapped to the annotated reference assemblies created above using the Geneious Mapper in Geneious Prime [54]. Each replicate was assumed to represent a pool of genetically diverse lineages as opposed to a single clonal organism [55]. Synonymous and non-synonymous variant calling was conducted in Geneious with default settings using the bacterial genetic code and a minimum variant frequency of 0.25 and at least 50-fold coverage [56], excluding those sections of the genome outside of coding sequences. The minimum read depth for variant SNP calls was 12×, a stricter read depth criterion than has been used in comparable studies [57–59]. Annotation data for convergent mutations were recovered using NCBI Batch Entrez Search [60].

### (g) Determining the strength of selection, mutation rate and parallel evolution among experimental lineages

We calculated selection strength as *pN*/*pS*, the ratio of non-synonymous (*pN*) to synonymous (*pS*) polymorphisms within each experimental lineage relative to the ancestral genome [61–63]. *pN*/*pS* ratios can be affected by effective population size [64,65]; however, given we have accounted for starting population size (by using 50 μl for each transfer) and for the number of experimental generations, we are reasonably sure that *pN*/*pS* ratios reflect levels of selection. Generalized linear models with a Gamma error structure were used to test the effects of species, rifampicin-resistance phenotype, selection temperature and their interactions on *pN*/*pS*, as above. When rifampicin resistance was found not to significantly affect *pN/pS*, susceptible and resistant lineages for each species were collated. One-sample *t*-tests were used to determine whether *pN*/*pS* ratios differed significantly from neutrality (i.e. mu = 1), and two-sample *t*-tests were used to determine whether different temperatures incurred different levels of selection. Second, we used *pS* to infer mutation supply—the numbers of mutation present in the population—in each strain [66,67]. Analysis of *pS* values differed from that used for comparing *pN*/*pS* across treatments in that a linear model was used to analyse overall variable interactions, and only two-sample *t*-tests were used to determine whether pS differed significantly within a species by temperature.

To test whether mutations accumulated from selection rather than drift [66], experimental lineages were examined for evidence of convergent evolution. Variants in each protein present in more than half of the lineages (i.e. three or more) in a given treatment category within each species were considered convergent and therefore taken as evidence of directional selection [68]. The percentage of reads containing the ancestral read and the percentage containing the variant SNP were also examined [69]; complete replacement of an ancestral SNP was taken as evidence that the SNP had undergone selective sweeps [70]. Genes showing signs of convergent evolution in response to temperature had their functions based on Clusters of Orthologous Genes (COG) pulled from the NCBI database and compared [71].

## 3. Results

### (a) Differences in growth rate and productivity across different temperature conditions in mesophilic and psychrophilic *Bacillus* species

**Figure 1.**
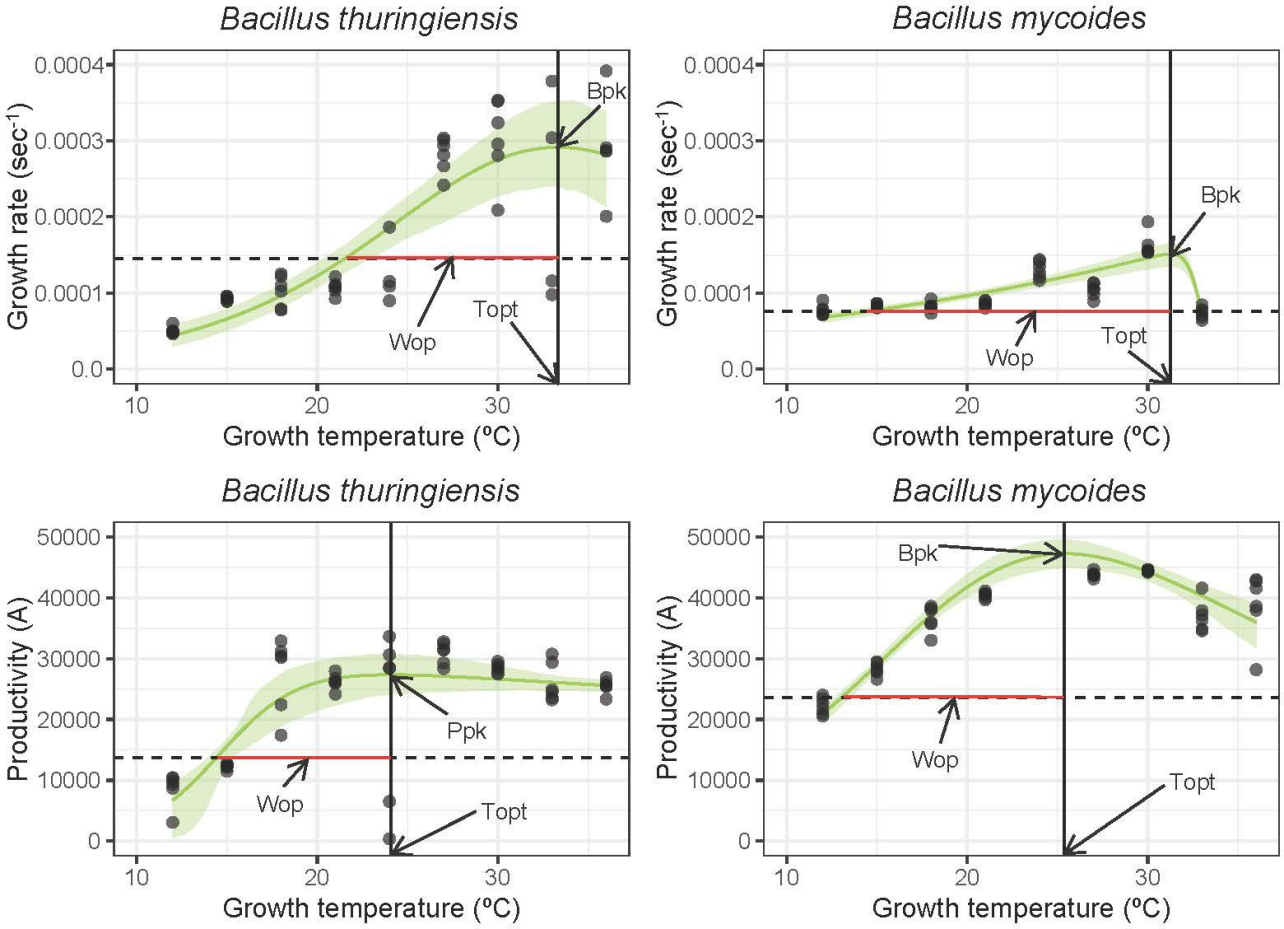
The relationship between growth rate (*r*)/productivity (A) and temperature in *B. thuringiensis* and *B. mycoides*. Thermal performance curves (TPCs) were quantified by the Sharpe–Schoolfield model as used previously [8] and shown here using the green lines with residual bootstrapped confidence intervals. These were used to extract the optimum growth temperature (*Topt*; 33.3°C and 31.26°C for the growth rate of *B. thuringiensis* and *B. mycoides*, and 24.23°C and 25.47°C for the productivity of *B. thuringiensis* and *B. mycoides*, respectively), the maximum growth rate/productivity (*Bpk/Ppk*) and the operational thermal niche width (*Wop*). Dots indicate productivity and growth rate values for growth curves of replicate cultures at each temperature.

In this study, we aimed to determine whether psychrotolerant and mesophilic bacterial strains showed different constraints to thermal adaptation. Thermal performance curves confirmed that *B. mycoides* and *B. thuringiensis* are psychrotolerant and mesophilic, respectively, and exhibit different thermal patterns of growth rate and productivity. Both species have optimum growth rates at temperatures around 30°C, at 31.26°C (95% CI: 31.24−31.39) and 33.3°C (95% CI: 30.43−39) for *B*. *mycoides* and *B. thuringiensis*, respectively. However, the operational thermal niches for these strains are quite different, with that of *B. mycoides* being over 5°C larger than that of *B. thuringiensis* (16.88°C compared with 11.77°C; figure 1). *B. mycoides* also shows a different curve shape, with an extended operational niche below the optimum temperature but a decreased operational niche about it (figure 1). Productivity shows similar differences, with optimum productivity achieved at 25.47°C (CI: 24.81, 26.10) and 24.23°C (CI: 21.24, 30.00) for *B. mycoides* and *B. thuringiensis*, respectively, with *B. mycoides* again having a wider operational thermal niche (12.26°C compared with 9.25°C in *B. thuringiensis*) (figure 1).

### (b) Evolved *B. thuringiensis* and *B. mycoides* lineages show significant thermal specialization

**Figure 2.**
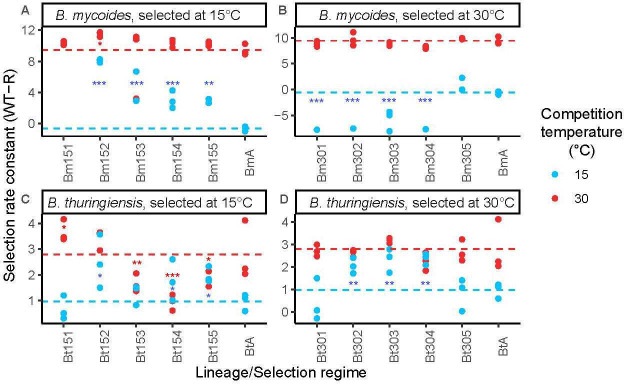
Lineage-level fitness changes after selection at 15°C (*A* and *C*) and 30°C (*B* and *D*) in *B. thuringiensis* and *B. mycoides*. Fitness is measured against rifampicin-resistance-marked ancestral strains as the selection rate constant. Dashed lines indicate ancestral WT fitness at each competition temperature; comparing the measure points to the dashed lines can tell whether the fitness of evolved lineages is higher or lower than that of the ancestor; a selection rate constant of 0 indicates no difference in fitness between competed strains (note than fitness was generally >0 as all evolved lineages were competed against rifampicin-resistant ancestors). Confidence intervals around the mean selection rate constant for ancestral strains were 1.30 and 0.37 for *B. thuringiensis* at 30°C and 15°C, respectively, and 0.81 and 0.38 for *B. mycoides* selected at 30°C and 15°C. Data points show the independent competition assays conducted between the ancestor strain and the evolved culture. Asterisks indicate lineages that are significantly different from their ancestor:p-values are represented by asterisks; *<0.05, **<0.01, ***<0.001.

We hypothesized that each strain would be constrained in their ability to adapt to new temperatures. In particular, we predicted that *B. mycoides* would experience adaptational constraint above its thermal optimum and *B. thuringiensis* would experience adaptational constraint below its thermal optimum. Overall, competitive fitness supported this hypothesis in relation to *B. mycoides*; here, fitness changes were dependent on the temperature in which it had adapted (selection treatment X competition temperature interaction *χ*^2^ = 17.65, d.f. = 2, *p* < 0.001, figure 2*A*). The majority of *B. mycoides* lineages showed improved competitive fitness at 15°C after selection at 15°C (Bm152, Bm153, Bm154 and Bm155, *p* < 0.01, figure 2*A*). However, *B. mycoides* could not adapt well to the higher temperature regime: 30°C selected lineages did not differ from their ancestor at all (*p* > 0.05) (figure 2*B*). There was also evidence for temperature-specific trade-offs in adaption, as selection at 30°C led to loss of fitness when grown at 15°C (Bm301, Bm302, Bm303, Bm304, *p* < 0.001) (figure 2*B*).

For *B. thuringiensis,* results were less clear-cut: selection temperature did have an overall impact on fitness (*χ*^2^ = 23.82, d.f. = 1, *p* < 0.001), but there was no interaction with competition temperature (*χ*^2^ = 0.46, d.f. = 2, *p* = 0.77). There was more between-lineage variation in response to selection in *B. thuringiensis* (figure 2*D*). Some of this can be interpreted as temperature-dependent trade-offs, three lineages selected at 15°C showed significant decreases in fitness at 30°C compared with their ancestor (Bt153 = −1.14, Bt154 = −1.86, Bt155 = −0.97, *p* < 0.02) (figure 2*C*).

### (c) Psychrotolerant strains did not experience the same thermal constraints as mesophiles, and showed different physiological responses to selection

**Figure 3.**
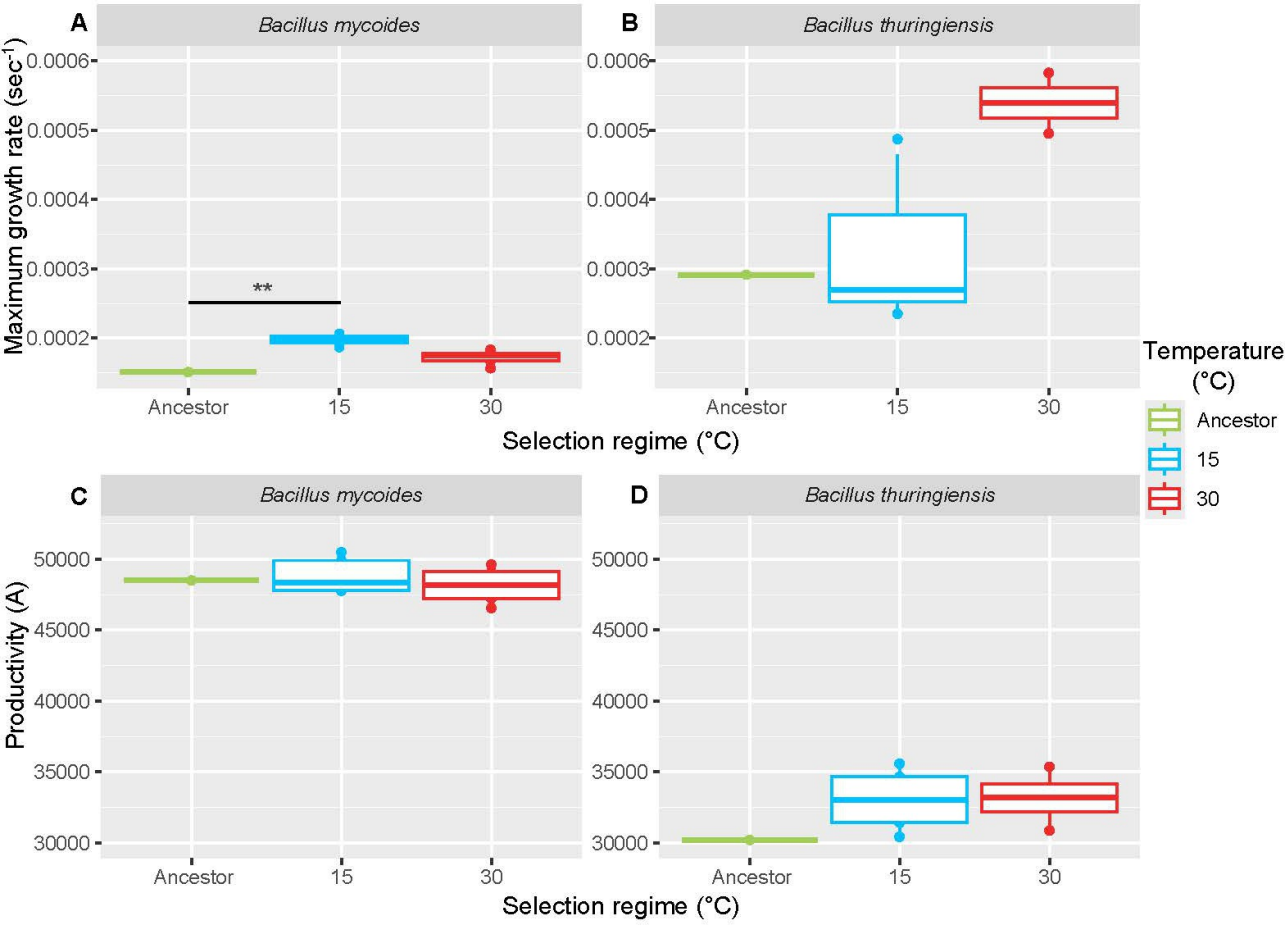
Effects of selection on growth rate (*A*, *B*) and productivity (*C*, *D*) in *B. thuringiensis* and *B. mycoides*. ‘Perfect adaptation’ predicts relatively invariant peak growth rate or peak productivity at thermal optima for strains adapted to different temperatures. Thermal optimum was calculated using the Sharpe–Schoolfield model [8].

We also used thermal performance curves to test how selection regimes had affected evolved strains relative to ancestors. This allowed us to test the hypothesis of perfect adaptation [8] which posits that minimal enzymatic constraints allow bacteria to adapt perfectly to new temperature conditions [4]. Due to high variability among replicates, we could not resolve TPCs for growth rate performance for three lineages of *B. thuringiensis* (Bt430, Bt315 and Bt515) and two *B. mycoides* lineages (Bm415 and Bm515). One outlier (Bt230) was also removed from the productivity analysis, due to a large amount of skew in the data that caused the TPC to give erroneously high *Topt* and *Wop* values (electronic supplementary material, figure S1). As with competitive fitness, *B. mycoides* lineages that were adapted to 15°C showed improved growth rates relative to ancestors (figure 3*A*; *F*_1,6_ = 16.38, *p* < 0.01). While *B. thuringiensis* lineages were tested, those adapted to 30°C (but not those adapted at 15°C) showed a trend in the direction of higher growth rate than their ancestor, but the difference was not significant (*F*_1,5_ = 6.09, *p* = 0.057) (figure 3*B*). In comparison, neither *B. thuringiensis* or *B. mycoides* lineages showed different levels of productivity from their ancestors after adaptation at either temperature (*p* > 0.24) (figure 3*C*,*D*).

**Figure 4.**
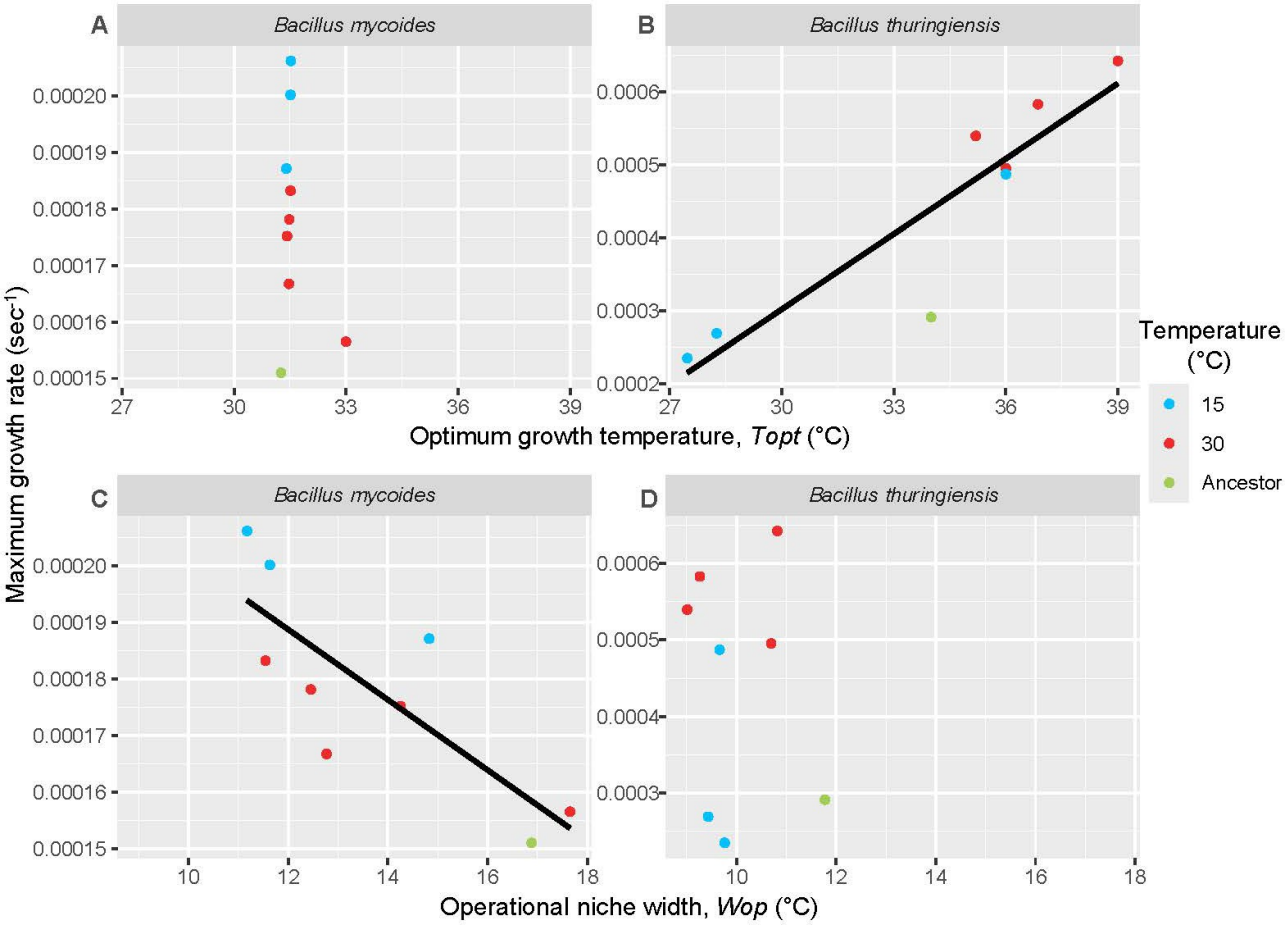
Effects of selection on growth rate, optimum temperature (*Topt*; *A* and *B*) and operational thermal niche width (*Wop*; *C* and *D*) in *B. mycoides* and *B. thuringiensis* lineages. The ‘HiB’ hypothesis predicts that optimum growth rate and productivity at thermal optima will correlate positively with optimum growth temperature, whilst a ‘specialist/generalist trade-off’ predicts that increases in growth rate or productivity will correlate with decreases in thermal niche width. Thermal optimum and thermal niche width were calculated for each lineage separately, using the rTPC and nls.multstart packages in R [43] and the Sharpe–Schoolfield model [8]. Lines are shown for significant relationships only.

We tested the HiB hypothesis, which predicts that the maximal growth rate at thermal optimum (*Bpk*) would correlate positively with the optimum growth temperature (*Topt*). *B. mycoides* lineages showed no significant correlation between maximal growth rate and *Topt*, a rejection of the HiB hypothesis (*χ*^2^ = 27.32, d.f. = 2, *p* = 0.36) (figure 4*A*). *B. thuringiensis* lineages showed a significant positive correlation between *Bpk* and *Topt*, in support of the HiB hypothesis (*χ*^2^ = 27.78, d.f. = 2, *p* < 0.001), with lineages selected at 30°C having higher growth rates than those selected at 15°C (*p* < 0.001) (figure 4*B*). We also tested the prediction of the ‘specialist/generalist trade-off’ hypothesis by testing the correlation of *Bpk* with operational thermal niche width (*Wop*). Here, we found a strong correlation among *B. mycoides* lineages (*χ*^2^ = 12.45, d.f. = 1, *p* < 0.001, figure 4*C*), but not among *B. thuringiensis* lineages (*p* = 0.75) (figure 4*D*).

When productivity (*Ppk*) was examined, it did not significantly correlate with either optimum temperature or thermal niche width in all species, with one exception; there was a significant positive correlation between *Ppk* and optimum temperature among *B. thuringiensis* lineages (*χ*^2^ = 9.48, d.f. = 1, *p* < 0.01, electronic supplementary material, figure S2). Productivity differed between temperature regimes in both species, with higher productivity after selection at 30°C in *B. thuringiensis* (*χ*^2^ = 6.08, d.f. = 2, *p* = 0.047*) and higher productivity after selection at 15°C in *B. mycoides* (*χ*^2^ = 10.02, d.f. = 2, *p* < 0.01**, electronic supplementary material, figure S2).

We tested whether fitness differences could be explained by variation in cell volume, as adaptation to warmer temperatures is sometimes accompanied by decreases in cell volume, which would not be detected in competition experiments [72,73]. We specifically looked for differences in lineages evolved at 30°C. We found some evidence for increases in cell volume in the *B. thuringiensis* evolved lineages (*F*_5, 247_ = 3.14, *p* = 0.009) driven by changes in lineages 1 and 4 (electronic supplementary material, figure S3). However, effect sizes were modest, with an increase in cell size of around 17% in lineage 4. In *B. mycoides*, we also saw variation in cell sizes in evolved lineages, but here four lineages produced slightly smaller cell sizes than their ancestor (*F*_5, 266_ = 10.6, *p* < 0.0001). Cell size in ancestral strains did not differ between species (*F*_1, 113_ = 1.27, *p* = 0.26).

### (d) Temperature adaptation in *Bc* was associated with parallel evolution and differed depending on strain

**Figure 5.**
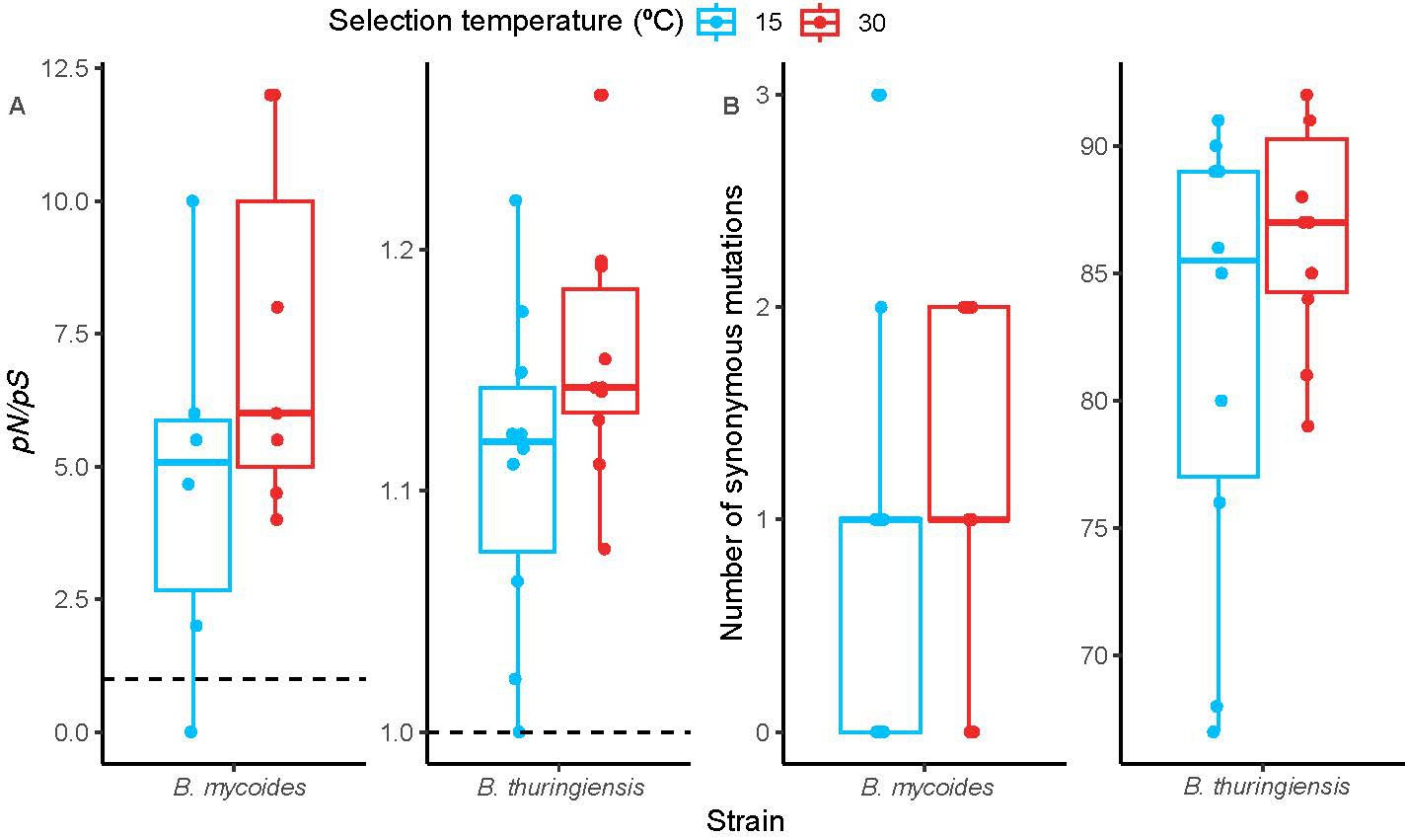
Variation in selection strength and mutation rate supply by strain and by selection regime. (*A*) *pN*/*pS* of evolved lineages as a proxy measure of selection strength [61,62]. (*B*) The number of synonymous mutations (*pS*) as a proxy measure of mutation rate supply [67]. Boxplots show medians, the first and third quartiles, and interquartile range. *Y*-axis values for each species differ due to the number of polymorphisms found in lineages belonging to each species, with fewer SNPs found in *B. mycoides* lineages than in *B. thuringiensis* lineages.

We explored if observed temperature-specific fitness changes were caused by differences in selection strength or mutation supply at different temperatures. First, we used the ratio between non-synonymous and synonymous mutations (*pN*/*pS*) as a proxy for selection strength. Here, we used both WT and rifampicin-resistant lineages, as these were not significantly different from each other (Likelihood ratio test; d.f. = 1, *χ*^2^ = 0.29, *p* = 0.59). All treatments showed *pN*/*pS* ratios greater than 1, suggesting directional selection (one-sample *t*‐test; *p* < 0.05). Crucially, *pN*/*pS* ratios did not significantly differ between selection regimes in either *B. thuringiensis* lineages or *B. mycoides* lineages (*t* = 1.6639, d.f. = 18, *p* = 0.11; *t* = 1.44, d.f. = 11, *p* = 0.18, respectively) (figure 5*A*); *pN*/*pS* was, however, higher for *B. thuringiensis* (species effect: Likelihood ratio test, *χ*^2^ = 46.2, d.f. = 1, *p* < 0.001).

Second, we used the number of synonymous polymorphisms (*pS*) to infer mutation rate in each strain [66,67]. As above, rifampicin resistance did not affect *pS* (Likelihood ratio test; d.f. = 1, *χ*^2^ = 0.29, *p* = 0.59) and so analyses were based on both WT and rifampicin-resistant lineages. The number of synonymous polymorphisms was not affected by selection temperature in either *B. mycoides* lineages (Wilcoxon rank sum test with continuity correction; *W* = 56, *p* = 0.37) or *B. thuringiensis* lineages (Welch two sample *t*‐test with unequal variances; *t* = 1.39, d.f. = 13, *p* = 0.19) (figure 5*B*). Mutation rate differed by species, with a higher number of synonymous mutations among *B. thuringiensis* lineages than among *B. mycoides* lineages (Likelihood ratio test; *χ*^2^ = 137, d.f. = 1, *p* < 0.001).

### (e) SNP analysis uncovered genome-wide changes linked to thermal adaptation

**Figure 6.**
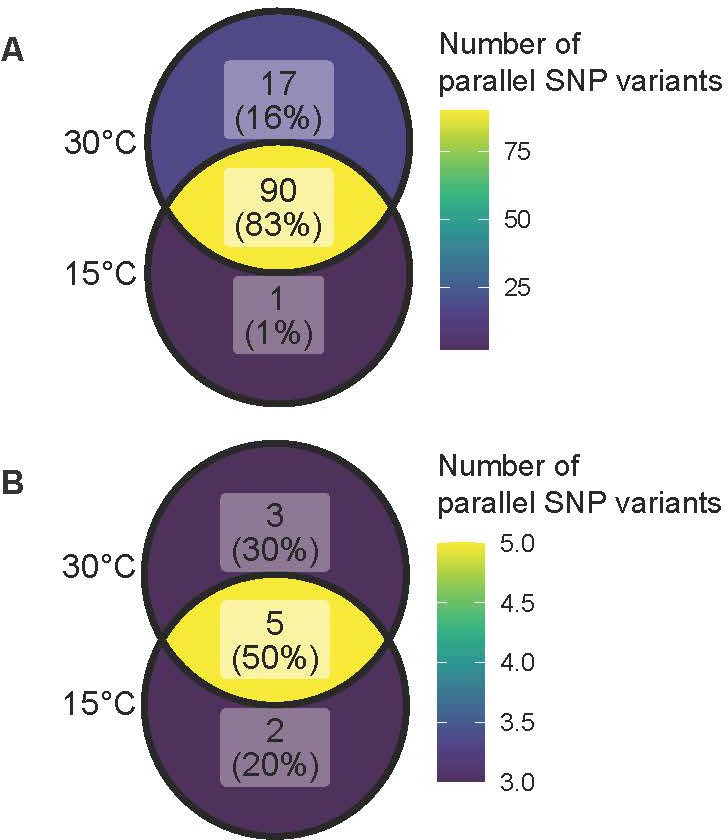
Venn diagrams showing the numbers of non-synonymous SNPs hypothesized to undergo parallel evolution in different selection regimes. Genes were considered to have undergone parallel evolution if they showed new SNP variants in more than half of lineages from the same treatment. (*A*) Parallel SNPs among *B. thuringiensis* lineages, (*B*) parallel SNPs among *B. mycoides* lineages.

Within the lineages descended from the *B. thuringiensis* ancestor, 108 protein-coding regions showed non-synonymous SNP variants that appeared in at least three lineages within at least one treatment, which we took as evidence of convergent evolution [68]. Of these, 83.3% (90/108) were present in both temperature treatments, suggesting they could play a role in adaptation to the media; 15.7% (17/108) and 0.9% (1/108) of non-synonymous SNPs showed changes in the 30°C and 15°C treatments, respectively. The convergent non-synonymous SNPs following selection at 15°C mapped to flagellin C (AGE77472.1), whilst the convergent SNPs at 30°C included the transcription factor MerR (AGE79104.1), an ABC transporter permease protein (AGE78365.1) and chaperonin groL (AGE75831.1) (electronic supplementary material, data 1). SNP variant analysis also identified non-synonymous changes within the *rpoB* gene associated with rifampicin resistance in all four resistant *B. mycoides* lineages [74] (figure 6), which indicates that the methods used in this study can detect real genetic variation associated with phenotypic differences.

Convergent changes were also found within the *B. mycoides* lineages; of the SNPs in 41 unique genes, 10 showed changes in multiple lineages in the same selection regime, with three convergent SNPs in the 30°C treatment (within a hypothetical protein, microbial collagenase (EC 3.4.24.3) and a transcriptional regulator from the AcrR family), while two genes showed parallel SNP variants in the 15°C treatment (polyketide synthase modules and related proteins, and 3-oxoacyl-[acyl-carrier-protein] synthase, KASII (EC 2.3.1.179)) (figure 6).

*B. thuringiensis* and *B. mycoides* lineages showed changes in genes with similar functions after selection at 30°C; COG functional analysis of the gene sets indicated that after selection at 30°C, both strains showed SNP variation in genes with COG categories associated with post-translational protein modification (O), transcription (K) and phages (X). However, responses to selection at 15°C differed between species, with *B. thuringiensis* showing changes in genes associated with cell motility (COG category N) and *B. mycoides* showing changes to secondary metabolite and lipid transport (Q and I, respectively) (electronic supplementary material, data 2).

## 4. Discussion

Across species from microbes to insects, rates of adaptation are heavily temperature dependent [4,10,15,16]. It is already accepted that climate change affects pathogens and may contribute to the emergence and re-emergence of disease [1,75]. Therefore, understanding how temperature affects adaptation to novel conditions in different species of bacteria is crucial. In particular, thermal performance curves of mesophiles commonly support the ‘HiB’ and ‘specialist-generalist trade-off’ hypotheses, i.e. that growth rate at thermal optima positively correlates with optimum temperature and negatively correlates with thermal niche width, respectively [6–8]. In this study, the mesophile *B. thuringiensis* showed patterns consistent with the expectations of the ‘HiB’ hypothesis, with a strong correlation between temperature and optimum growth rate. The resequencing data and a strong trend in growth rate data pointed towards temperature-dependent adaption, with more parallel SNP changes at 30°C; however, these data were not supported by measures of competitive fitness. Between-lineage variability, the low power derived from small sample sizes and using low fitness rifampicin-resistant competitors, and the inability to fit thermal performance curves to all evolved lineages are limiting; however, this disparity could not be explained by changes in cell size in either species, which were relatively small compared with previous studies [76] and which were not associated with significant increases in fitness in the case of *B. mycoides* (figures 2 and 3). This suggests that fitness changes and operational niche width is driven primarily by thermal sensitivity, which is largely independent of cell size [8].

Phenotypic plasticity may explain some variation between populations selected at the same temperature; the effect of phenotypic plasticity on bacterial evolution is only beginning to be understood [77]. Assessing plasticity is challenging following experimental evolution, as evolved lineages will contain multiple genotypes [78,79] and the length of such experiments allow large amounts of time for plasticity to affect phenotypes [80]. To reduce experimental noise, we used standard acclimation conditions at the beginning of the experiments to standardize physiology. Consequently, while phenotype plasticity may have contributed to observed phenotypes in our experiment, we did not attempt to measure phenotype plasticity.

The psychrotolerant *B. mycoides* did not show increased competitive fitness after selection at a temperature close to its thermal optimum. Correlations between growth rate or productivity with temperature were inconsistent with the ‘HiB’ hypothesis, but growth rate patterns did support the specialist-generalist trade-off hypothesis. Furthermore, there was evidence that *B. mycoides* was better adapted—and better able to adapt—at lower temperatures, showing losses of fitness following selection at 30°C and improved growth rate following selection at 15°C; almost the exact opposite patterns shown by *B. thuringiensis*. In summary, our measurements of competitive fitness showed that *B. mycoides* was better able to adapt to its ‘preferred’ temperature regime, while results were more equivocal for *B. thuringiensis*. This supports our original hypothesis that psychrotolerant bacteria show constraints in adapting to warmer temperatures that are not faced by mesophiles.

Thermal optimum—in terms of growth rate—did not change following selection at either temperature for *B. mycoides* (figure 4*A*). This contrasts with predictions that thermal optima of growth rate evolve quickly under different conditions [16]. Despite this, response to selection can be seen in changes to operational thermal niche width (figure 4*C*) and in convergent SNP variants. Thermal optimum is not the only phenotypic change that can increase fitness at different temperatures; in particular, others have noted the role of enzymes and other conformational changes to grow at new temperatures [21]. *B. mycoides* is defined as having a different thermal niche not due to differing in thermal optimum from *B. thuringiensis*, but because it has the ability to grow at 7°C when *B. thuringiensis* does not, an ability linked to variation in a specific cold-shock protein gene, *CspA* [11]. As we saw no SNP changes in this gene during our analysis, we can assume that this adaptation— as with growth rate—is not the only mechanism by which *B. mycoides* might respond to changes in temperature conditions.

Previous research has shown that classic metabolic theory holds for mesophiles, but not for thermophilic microbes adapted to extreme heat [16]. This may be due to specific adaptations developed by thermophiles to tolerate high temperature; these adaptations can involve regulatory functions and improved efficiency of bacterial growth [33,81,82]. Convergent genetic changes identified during adaptation to 15°C among *B. mycoides* lineages are consistent with adaptation to cooler temperatures. We identified novel SNPs in genes linked to regulatory functions such as the *acrR* transcriptional regulator, and a mutation in polyketide synthase modules, the latter of which has previously been linked to cell membrane fluidity via fatty acid biosynthesis [21,83]. Additionally, COG functional enrichment suggested that adaptive responses to 15°C—but not to 30°C—differed between *B. thuringiensis* and *B. mycoides* lineages. An avenue of investigation that can link specific genetic changes to phenotypic outcomes—using genome-wide association studies (GWAS) for instance [84]—would be useful for putting these findings in their broader context and indicate how widely distributed thermal niche constraints are within the *B. cereus* group.

It is also important to consider how response to selection can affect fitness parameters other than growth rate. Growth rate is the growth parameter that is most easily investigated and forms the basis of most theories involving reaction rates [12,85–90]. However, productivity may be a more comprehensive measure of bacterial performance, incorporating carrying capacity and starting population size as well as growth rate [37]. We found that species was significantly associated with growth rate and productivity, but with marked differences; the correlations observed between growth rate and optimum temperature or niche width in *B. thuringiensis* and *B. mycoides*, respectively, did not appear when productivity is used (figure 4*B*,*C*); however, we found that productivity changes did vary between species in different thermal treatments, with *B. mycoides* having increased productivity after selection at 15°C and *B. thuringiensis* having increased productivity at 30°C (electronic supplementary material, figure S2). While we lack the statistical power to be certain, this suggests that *B. mycoides* may be adapting to temperature conditions in subtly different ways to *B. thuringiensis*. This highlights the importance of considering multiple growth parameters when determining levels of thermal adaptation constraint, to ensure that different solutions to get around such constraints are not missed [91].

This study poses a question that has not yet been properly answered; how do bacterial strains adapt to decreases in temperature, i.e. what determines the upper and lower limits of thermal niches for mesophilic and psychrotolerant species? Here, we considered adaptation to a temperature below the optimum for both species. If bacterial strains are highly constrained in their adaptation to new temperatures based on thermal dynamics, then one might expect adaptation to low temperatures to be slow [8]. While that may be true in some contexts, we showed that, after controlling for generation time, adaptation to temperatures below optima can vary with thermal niches, suggesting not all strains are similarly constrained. This is supported by the association between phylogeny and thermal traits in natural habitats such as soil; cold-adapted bacteria are incredibly diverse and thermal adaptation has been found to be associated with latitude and phylogeny in more than one species [19,92]. Whilst thermal phenotypes might evolve rapidly under certain conditions [15], adaptation to temperature appears constrained, with phylogeny-dependent mechanisms and degrees of adaptation.

In conclusion, this study shows that not all bacteria follow the expectations of classic metabolic theory. Mesophiles and psychrotolerant bacteria showed different responses to selection in terms of fitness change, phenotypic response and genes linked to adaptation. Importantly, the difference in the direction of constraints observed in this study suggest that mesophiles could readily adapt to warmer temperatures and psychrotolerant species to cooler temperatures, but would struggle to adapt under the opposite conditions. This partially explains why thermal biology is a potent force driving bacterial speciation; phylogenetic separation is often closely associated with thermal niche [33,92] and signatures of thermal adaptation can be found genome-wide [33], meaning that temperature-dependent selection likely acts on a large number of genes, creating potent forces for genetic and evolutionary coherence.

## Data Availability

All genetic information is available through NCBI, via the Bioproject PRJNA826440. All other data and code necessary to recreate the results and figures in this manuscript are deposited on Dryad [93]. Supplementary material is available online [94].
